# Design and Analysis of an Active Reflection Controller That Can Reduce Acoustic Signal Refer to the Angle of Incidence

**DOI:** 10.3390/s21175793

**Published:** 2021-08-28

**Authors:** Joo Young Pyun, Beom Hoon Park, Young Hun Kim, Yeong Bae Won, Hak Yi, Jeong-Min Lee, Hee-Seon Seo, Kwan Kyu Park

**Affiliations:** 1 Department of Convergence Mechanical Engineering, Hanyang University, Seoul 04763, Korea; jooyoungpyun@hanyang.ac.kr (J.Y.P.); pbh128@hanyang.ac.kr (B.H.P.); jason401@hanyang.ac.kr (Y.H.K.); 2 Department of Mechanical Engineering, Kyungpook National University, Daegu 41566, Korea; wyp1028s@knu.ac.kr (Y.B.W.); yihak@knu.ac.kr (H.Y.); 3 Maritime Technology Research Institute, Agency for Defense Development, Changwon 51682, Korea; leemin@add.re.kr (J.-M.L.); hsseo@add.re.kr (H.-S.S.)

**Keywords:** low-frequency transducer, piezoelectric material, angle of incidence

## Abstract

Techniques for reducing the reflection of acoustic signals have recently been actively studied. Most methods for reducing acoustic signals were studied using the normal-incidence wave reduction technique. Although the technique of canceling an object from the normal incidence wave is essential, research on reducing acoustic signals according to the angle of incidence is required for practical applications. In this study, we designed, fabricated, and experimented with an active reflection controller that can reduce acoustic signals according to the angle of incidence. The controller consists of a transmitter on one layer, a receiver sensor on two layers, and an acoustic window on three layers. To reduce the reflected signal, a combination of the time delay and phase was applied to the controller to minimize the acoustic signal by up to −23 dB at an angle of 10°. A controller array simulation was performed based on the results of a controlled experiment. In conclusion, our proposed controller can reduce acoustic signals according to the angle of incidence, which makes it suitable for many applications.

## 1. Introduction

Research has been conducted on reducing or scattering acoustic signals as a countermeasure against underwater acoustic detection systems. Wedge shapes, anechoic coatings, and met surfaces have been developed to reduce acoustic signals [1,2,3]. The wedge shape was developed using butyl rubber, and the technique of annihilating sound waves was applied to it. Designing the wedge shape involves relatively fewer design steps compared to those in other methods; however, attaching it to a moving object is ineffective for reducing low-frequency signals. Furthermore, the low frequency exhibits a long wavelength. Therefore, the thickness of the wedge-shaped sound-absorbing material must be considerably thicker to reduce the sound absorption performance. Therefore, if the absorber is attached to a moving object, it may affect the movement of the object owing to the load of the thick sound-absorbing material [4,5]. To design an anechoic panel made of baffle, aluminum backing, and rubber, a study was conducted to vary the tip angle at the end of the panel in a specific frequency range [6]. To absorb the acoustic signal in the low-frequency region, the samples were compared to select a suitable sound-absorbing structure [7,8].

For efficient absorption, an acoustic panel was constructed as follows: It comprised an anechoic coating, a steel plate, and a rib. The steel plate and ribs assist in lowering the sound absorption frequency band [9]. Moreover, the sound absorption performance was demonstrated for various frequencies using polydimethylsiloxane (PDMS) and pores. If it is composed of a soft material, it applies a relatively minor load to the movement of the moving body when attached to a moving body. However, the composed materials seem to lack durability in water [10]. Another method is to use an Alberich anechoic coating. The anti-reflective coating had a pore morphology. Owing to the shape of the pores, sound waves were scattered, creating a sound-absorbing effect. The anechoic layer exhibits excellent sound absorption properties; however, it works well only in a specific frequency band, depending on the pore shape. In addition, to increase the sound absorption bandwidth using anechoic coatings, different pore sizes must be designed [11,12,13]. In a study based on a viscoelastic material, a certain method was adopted to increase the sound absorption performance according to the material of the inner core and angle of the ellipsoidal. To dissipate the sound, the inner core material comprised steel and aluminum; the sound absorption performance was better in the steel core [14]. Since a considerable time is required to design and experiment with coating technology, model optimization has been performed for a suitable specific frequency range [15]. The sound absorption characteristics for each frequency were identified after constructing an array rather than a single structure. The sound absorption effect was remarkable when the coated and uncoated samples were compared [16]. 

When applied to a low frequency to demonstrate the sound absorption, a limit is observed to the thickness of the structure. In addition, a study was conducted to broaden the sound absorption bandwidth based on metamaterials at low frequencies. In general, four factors must be considered when designing a metamaterial unit. Viscoelastic matrix rubber, cavities, oscillators, and backing materials are present. The thicker the backing material in this structure, the higher the sound absorption performance [17]. In addition to sound-absorbing materials, woodpile absorbing materials have been examined in recent studies. After coating the steel rod with polyurethane, it was continuously laminated and studied. The aforementioned structure has a diameter of 5 mm and can exhibit sound absorption performance in the range of 2.8–8.8 kHz [18]. Helical structures have been used in sound absorption studies. 

The sound absorption performance varies depending on the shape and material. The sound absorption performance was higher when the spiral was made of a rigid material rather than a soft material [19]. Another study using a meta structure technique employed two metal disks. The two metal disks were made of tungsten: the higher the density of the disk, the higher the sound absorption performance [20]. In general, an underwater vehicle can detect an object approaching from the outside. The design of such an underwater vehicle is essential, and optimized modeling is required to improve the driving force of the vehicle. Therefore, the shape of a moving body that can be applied to multiple frequencies for a given angle has also been studied [21]. Furthermore, a method has been proposed to reduce acoustic signals using a clocking technique [22,23].

Wedge shapes, anechoic coatings, and meta structures have been consistently used to reduce acoustic signals but have not overcome their thickness. Recently, ultrathin coatings have also been studied [24]. The ultrathin coating had a final thickness of 0.488 mm and comprised three layers. It consists of a multi-resonant unit, a PDMS layer containing air, and a copper plate. An attempt was made to widen the sound absorption bandwidth using multiple resonators and air, and the thickness and sound absorption performance were excellent. However, the durability was found to be low for long-term use in seawater. It can withstand high-pressure environments in the deep sea; however, it cannot be used for an extended period because the components are brittle in the deep sea. 

In addition to the passive sound-absorbing materials, studies on passive-active absorbers have been conducted. The disadvantages of passive sound-absorbing materials were highlighted at low frequencies; therefore, a study was conducted to insert an actuator inside the sound-absorbing material to absorb the sound. Based on a piezo actuator, the other layers were constructed using sound-absorbing materials. Although the sound-absorbing effect was compared by changing the thickness of the sound-absorbing material, it is difficult to apply it to the actual environment as only a theoretical analysis has been carried out [25]. An experiment was conducted in an anechoic room using the Filtered-x Least Mean Square (FxLMS) algorithm to determine the sound absorption effect of the passive-active panel. The experiment was conducted at a specific frequency, and the sound absorption was based on a cell located at the back of the panel [26].

Smart forms exist as passive-active structures. To apply it to low frequencies, the thickness of the passive sound-absorbing material becomes considerably thicker, and the weight and thickness increase according to the thickness. To overcome this, a relatively light smart foam study was conducted. The sound absorption performance of the smart foam made of polyvinylidene fluoride(PVDF) with a plexiglass cavity was tested, and the sound absorption coefficient was derived with or without control by placing the smart foam in the center, and evenly spaced microphones on both sides [27]. Another study compared smart-form-based control methods. When the adaptive feedforward and feedback methods were applied to the same smart form and compared, the feedforward method was more efficient. The feedback method has low efficiency because it is difficult to apply below a specific frequency. Because this method arranges microphones at regular intervals outside, there is a high possibility that the sound absorption efficiency can be normally derived only when the setting method is fixed [28]. After the appearance of the smart form structure, the noise was canceled by changing the connection method of PVDF, which is the actuator inside the smart form. In all three cases, original, parallel, and serial-parallel, we used a feedforward Least Mean Square(LMS) controller to minimize noise [29]. Smart form research has been continued until recently. Recently, the smart form had reduced noise based on the time delay and feedforward method [30].

Techniques related to active control rather than passive-active methods were also studied. There is an example of comparing three control techniques by placing 12 piezoelectric materials inside a smart shell. It is an excellent way to compare control methods by changing them. However, it is necessary to slightly alter the foam type, which has 12 elements arranged. The number of devices used is three [31]. Although control techniques and structural changes have been incorporated to increase the efficiency of sound-absorbing materials, prior studies have been conducted in air, not underwater [32,33,34]. Acoustic sound absorption characteristics are shown based on the active control in the water. The tonpilz transducer is input, the controller is composed of 1–3 piezoelectric composites, and the remaining two sensors are PVDF. A 30 dB echo reduction is possible at 4–11 kHz, but this is only possible for normal incident signals. Because all the receiving sensors exist independently outside, it is similar to arranging microphones in an array among studies conducted in the air, thus inhibiting a challenge to its application in a real environment. This is significantly complicated, and the maximum efficiency can be achieved only inside the tube [35]. Another previous study to reduce the acoustic signal inside the tube showed an echo reduction of more than 50 dB at 5.4 kHz. Although the sound absorption performance is outstanding at a specific frequency, the actuator sensor exists independently [36]. In addition to active control with a single unit, a study has been conducted on active noise control by implementing actuators as an array. When controlled with an array, the performance was superior to that of the passive absorber, but only for normal incidence [37]. 

In addition to normal incidence, the oblique incidence of the sound absorbers has been studied. Although it exhibited 94% sound absorption performance at 266–1500 Hz, it was performed up to 21° in an anechoic room [38]. Oblique incidence studies were also conducted on structures with anechoic coatings and steel backing. The sound absorption performance was compared according to the thickness of this structure, and only a theoretical analysis was performed without conducting an actual experiment [39]. A recent study on the oblique incidence of sound-absorbing materials derived reflection coefficient values at intervals of 15° from 0° to 60°. However, these are simulation results and not actual measurements [40].

Our study proposes a technique for reducing the acoustic signals by referring to the angle of incidence, in which the underwater controller and sensor are packaged together. A method that can reduce the acoustic signal according to the angle of incidence in water was derived by conducting an experiment. In our previous study [41,42], a study on normal incidence was performed, so we focused the study on the angle of incidence, except for normal incidence. The controller we have presented can be applied to an underwater vehicle when configured as an array.

## 2. Transducer Concept

**Figure 1:**
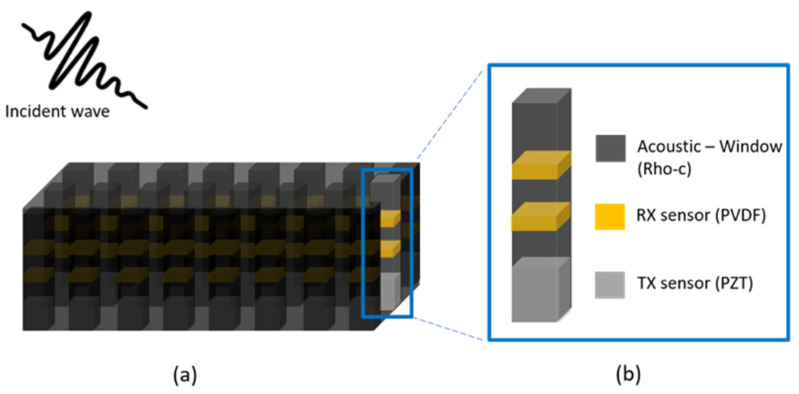
Concept of the transducer: (**a**) array type; (**b**) single transducer.

The proposed transducer, which can cancel the reflected signal, is shown in Figure 1. Owing to the nature of acoustic reflection, the angle of reflection is the same as the angle of the incidence wave. The reflected acoustic signal can be reduced using an active compensation signal from a transmitter(TX). The compensation signal can be calculated by measuring the two receiving sensors per unit. These compensation methods were presented in our previous work [42] concerning normal incidence waves. However, in the case of a non-normal angle of incidence, the transmitted acoustic signal is omnidirectional, and hence generates undesired acoustic signals in various directions. This issue can be addressed using an array structure of the transmitter. Here, single-channel transducers were arranged at 1/2 wavelength intervals to form an array, as shown in Figure 1a. If the transducer is configured as an array, then the acoustic signals can be efficiently reduced according to the angle of incidence. To focus on the main lobe, which increases the transmission efficiency of the transducer, the interval between elements was set to 1/2. 

A unit transducer constituting the array structure is shown in Figure 1b. The transducer consists of one layer of transmitter and two layers of receiving sensors. One layer of the transmitter reduces the acoustic signal, and the two-layer receiving sensor is configured to separate the incident and reflected waves. To design the transmission sensor, we proceeded as follows: First, the thickness of the piezoelectric material that resonated with the target frequency was selected. After selecting the target frequency, the material was selected. Lead zirconate titanate (PZT) was chosen as the piezoelectric material because of its decent transmitter sensitivity and material availability. The receiving sensor was PVDF, which has a wide bandwidth, can receive a broader area than piezoelectric materials, and is easier to handle. The PVDF receiving sensor was 110 μm thick and had a center frequency of 10 MHz. The transmitter and receiving sensors were composed of different materials, as shown in Figure 1b.

There is an acoustic window for coating and spacing between the transmitter and receiving sensors. This window was used to provide the space between two receiving sensors in positions separated by a quarter wavelength of 0.33 $f_{0}$, which is the target operating frequency. Elastomers, such as PDMS or Ecoflex, can be used for physical distancing. However, instead of these commonly used elastomers, Rho-C rubber was used in the unit because of its resistance to seawater and good acoustic impedance matching. The final model consists of a transmitter sensor on the first layer, the receiving sensor on the second layer, and an acoustic window on the third layer.

## 3. Simulation and Transducer Design

**Figure 2:**
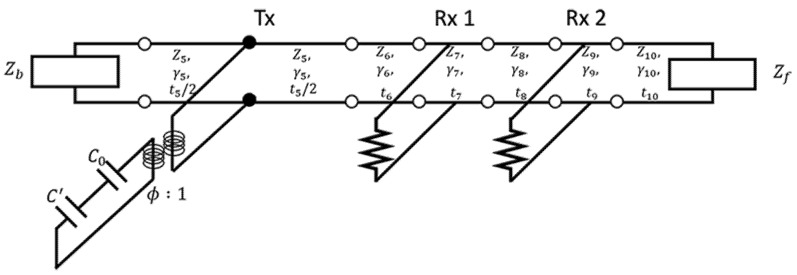
Simulation model [42].

To implement a transducer that controls reflection signals, the electrical characteristics and output pressure of a transducer must be predicted. To understand the electrical characteristics and output pressure of a transducer at the design stage, we analyzed it based on an analytical model. As shown in Figure 2, the model used for the analysis consisted of one layer of transmitter, two layers of receiving sensors, and three layers of acoustic windows [42]. 

This model acoustically transforms the mechanical part into a transformer. Our model was interpreted by considering the acoustic impedance *Z*, propagation constant *γ*, and thickness of the material *t*.

(1) $$c_{0}=\frac{\varepsilon A_{0}}{d}$$

(2) c′=−c0kt2 sinc (ωω0)

(3) $$\phi =\frac{k_{t}}{\sqrt{2f_{0}c_{0}Z_{c}}}sinc\left(f/2f_{0}\right)$$

The capacitance values were calculated using Equations (1) and (2). The transformer that acoustically changes the mechanical properties can be calculated using Equation (3). $c_{0}$ is the capacitance, and ε is the permittivity. $A_{0}$ is the area of the transducer, and *d* is the thickness of the transducer to be analyzed. $k_{t}$ is the coupling constant, and $f_{0}$ is the resonance frequency of the transducer [36,42].

(4) $$M_{i}=\left[\begin{array}{cc} cosh\gamma_{i}t_{i} & Z_{i}sinh\gamma_{i}t_{i}\;\\ \frac{1}{Z_{i}}sinh\gamma_{i}t_{i} & cosh\gamma_{i}t_{i} \end{array}\right],\;i=6,\;\cdots ,\;10$$

Each layer can be expressed as an *M* matrix, as shown in Equation (4). After substituting all material properties for each layer, each *M* matrix was multiplied. All the *M* matrices were multiplied.

The multiplied *M* matrix of each layer can be expressed using Equation (5) [36,42].

(5) $$M_{all}=M_{1}\times M_{2}\cdots M_{n}=\left[A\;B\;C\;D\right]$$

(6) $$Z_{e}=\frac{V}{I}=\frac{AZ_{F}+B}{CZ_{F}+D}$$

(7) $$v=\frac{-V}{AZ_{F}+B}$$

(8) $$V=F_{s}\frac{R_{s}}{Z_{f}\left(CR_{s}+A\right)+DR_{s}+B}$$

(9) $$P=v\frac{Z_{F}}{A_{0}}$$

Using Equation (6), we can derive the electrical impedance of the device. First, the resonance frequency of the device was determined by calculating the electrical impedance. Equation (7) allows the calculation of the surface velocity of the device. In Equations (6) and (8), *V* is the applied voltage. As shown in Equation (9), P is the transducer’s surface pressure and is derived from the velocity of the transducer surface, impedance of the transducer front, and area [36,42].

To design a transducer, the target frequency must first be determined. Accordingly, we set the target frequency as $f_{0}$ and selected the thickness of the piezoelectric material. 

**Figure 3:**
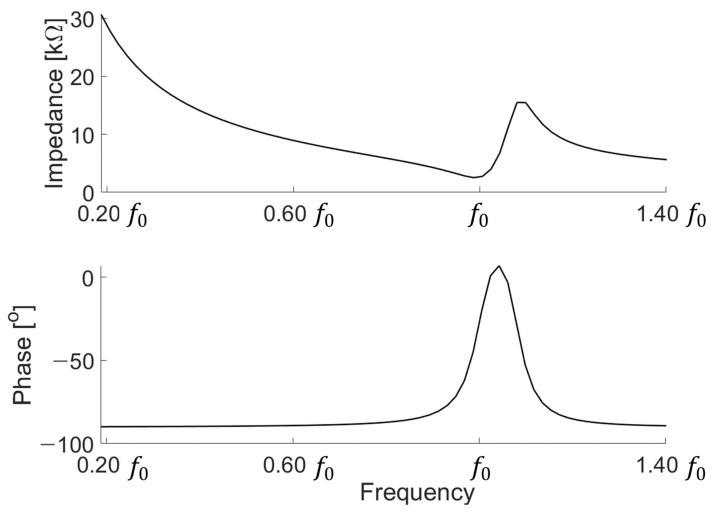
Electrical characteristic of the transducer.

The electrical characteristics of the transducer are shown in Figure 3. The figure also shows the simulation results, with the number of points from 0.20 $f_{0}$ to 1.40 $f_{0}$ as 500. Figure 3 shows that the device fits the target center frequency $f_{0}$.

**Figure 4:**
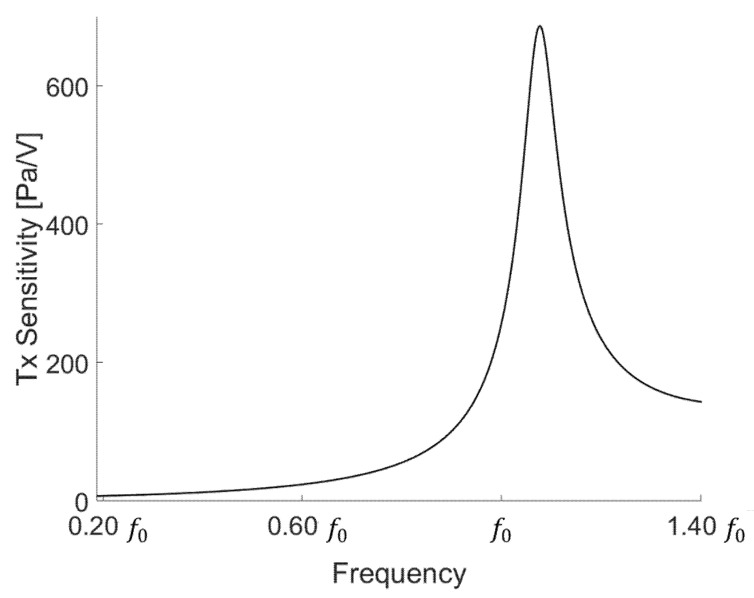
Transducer output pressure.

Figure 4 shows the output characteristics of the device designed at 0.20 $f_{0}$ to 1.40 $f_{0}$. The designed device has a maximum performance of 686.6 Pa/V at 1.06 $f_{0}$. Each transducer has a resonant frequency. At the resonance frequency, the transducer exerts its maximum output, but a ringdown phenomenon occurs. If the ringdown is severe, then it is difficult to determine the signal to be measured because it overlaps with the internal reflection of the tank and the reflection of the water surface. To avoid the ringdown phenomenon due to the narrow fractional bandwidth, we designed the target operating frequency to be 0.33 $f_{0}$, at which the non-resonance ringdown is relatively less than that in the resonance mode. 

Our controller was designed to reduce acoustic signals at 0.33 $f_{0}$. The two-layer receiving sensor maintains quarter-wave spacing to separate the incident and reflected waves. Because it was designed to control the acoustic signal at 0.33 $f_{0}$, the quarter wavelength of the receiving sensor was referenced to 0.33 $f_{0}$. The characteristics of the receiving sensor can be determined by considering the distance and acoustic window of the receiving sensor located at a distance of 1/4 wavelength. Based on the feedback system of the two receiving sensors, the sensitivity of the incident ($S^{+}$) and reflected acoustic signals ($S^{-}$) can be calculated using Equations (10) and (11).

(10) $$S^{+}=S_{B}e^{jkd}-S_{A}$$

(11) $$S^{-}=-S^{+}e^{-jkd}$$

where $S_{A}$ and $S_{B}$ are the receiving signals of the two receiving sensors. Two receiving sensors enable the separation of the incident wave and reflected signal, which are used in the compensation of the reflected acoustic signal [36,42]. 

## 4. Transducer Fabrication

We adopted a method of stacking PZT (PZT-5A, Piezo.com, Division of Mide Technology, Woburn, MA, USA) to implement a device with the target frequency. Thick piezoelectric materials are difficult to polish. Hence, the piezoelectric material was stacked and used at low frequencies. To stack the piezoelectric elements, we tried several methods and selected the most efficient methods. 

**Figure 5:**
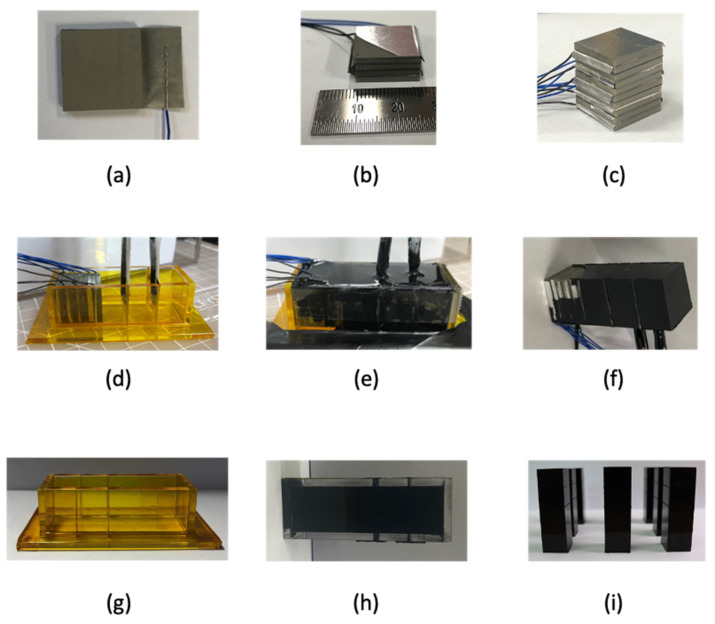
Fabrication flow: (**a**) device electrode connection; (**b**) stacking devices in two layers; (**c**) completed stacked device; (**d**) fixing the transmission and reception sensors inside the mold; (**e**) filling the acoustic window inside the mold; (**f**) completed a one-channel low-frequency transducer; (**g**) mold for making a rubber pillar; (**h**) cured Rho-C pillar; (**i**) completed Rho-C pillars.

We adopted the method of stacking using the Mylar tape. As shown in Figure 5a, a Mylar tape, wire, conductive epoxy (CW-2400, Chemtronics, Kennesaw, GA, North America), and low-viscosity epoxy (EPO-TEK^®^ 301, Epoxy Technology, Billerica, MA, USA) were used to laminate the PZT. In the case of the Mylar tape, the part where adhesive force exists is conductive. However, when the wire was attached to the Mylar tape without the addition of any other material, the signal was unstable. Thus, we used it after applying conductive epoxy when connecting the wire with the Mylar tape. Conductive epoxy was evenly coated on wires, which were attached to the Mylar tape. The conductive epoxy-coated wire was placed on the Mylar tape, folded in half, and pressed onto the back of the tweezer. 

As shown in Figure 5b, the next step is to repeat the process shown in Figure 5a. A low-viscosity epoxy was applied to laminate each layer of the piezoelectric plate. EPO-TEK^®^ 301, which is a low-viscosity epoxy and consists of agents A and B, was mixed at a weight ratio of 4:1. The mixed epoxy was placed in a vacuum chamber (stainless steel vacuum and degassing chamber, Thomas Scientific, Swedesboro, NJ, USA) for 10 min to remove bubbles existing between the mixed epoxies. To increase the adhesion of the piezoelectric element, we placed the piezoelectric element on a hot plate (HP 180D, MTOPS, Seoul, South Korea) at 65 °C, and the epoxy was placed in a vacuum chamber. 

The defoamed low-viscosity epoxy was applied to the piezoelectric material and laminated in two layers. Even if a low-viscosity epoxy was used, an epoxy layer was formed if it was not thinly applied. Hence, we used toothpicks to apply the epoxy as thinly as possible. After applying the epoxy thin layer, the process of wiping with a toothpick was repeated. The PZT was stacked with added weight of 100 g each. Slide glass and tweezers were used to align the stacked devices. One of the methods used for precise alignment involves placing the element on a hot plate set at 60 °C in advance. When laminated using a low-viscosity epoxy, if heat was not applied, then the piezoelectric plate would not align due to the epoxy. All the stacked devices were not stacked in the same direction. 

All the stacked devices are shown in Figure 5c. To check whether all stacked devices were properly driven, impedance was measured for each device layer, and was measured after connecting each layer to drive one. As shown in Figure 5c, the upper part consisting of the receiving sensor and acoustic window (Aptflex-F21, Precision Acoustics, Dorchester, Dorset, UK) was fabricated. We proceeded with two methods of connecting the receiving sensor, and between them, we chose the method with the highest yield for sensor manufacturing. The first method involves placing the receiving sensor inside the mold first and then connecting the electrodes, and the second method involves connecting the electrode to the receiving sensor and then putting it in the mold. The first method can minimize the exposure of the electrode from the outside. However, in this method, the receiving sensor is easily separated when removed from the mold. As shown in Figure 5d, we adopted the method that involved connecting the electrode to the receiving sensor and then putting it in the mold. When connecting the electrode to the receiving sensor, the wire is connected after fixing it, such that it is located at the edge of the receiving sensor. In addition, a low-viscosity epoxy was used on the receiving sensor, to which the electrode was connected to minimize the separation of the wire from the sensor. The receiving sensor has top and bottom surfaces coated with gold. When fabricating the sensor, a receiving sensor with a size of 12.9 × 12.9 λ is cut and used, but if the gold coating scratches even slightly during cutting, then the signal cannot be accurately received. When cutting the receiving sensor, we put a sound-absorbing material and then cut it with a knife. As shown in Figure 5e, after all the electrodes were connected, the transmitter and receiving sensors were placed inside the mold, and an acoustic window was poured. The acoustic window was composed of agents a and b and was used by mixing at a weight ratio of 3.35:1. The mixture was placed in a vacuum chamber for 10 min to remove bubbles generated during mixing. The acoustic window, from which the air bubbles were removed, was slowly poured into the mold. The acoustic window was susceptible to acetone in its pre-hardened state and was wiped with acetone if it flowed out of the mold. The acoustic window can be cured rapidly by applying a certain temperature; however, this was not possible because we had a receiving sensor inside the acoustic window. 

Our receiving sensor is vulnerable to heat, so we cured it at room temperature without applying heat. Figure 5f shows the primary completion of the sensor. In Figure 5f, all electrodes of the transmitter are exposed. A thin coating was applied. The defoamed acoustic window was coated with a cotton swab using the brush-touch method three times. Our device was designed to have an acoustic window on all sides of the main sensor. A mold for the acoustic window was made to attach the acoustic window to both sides of the sensor. The mold on each side of the acoustic window is shown in Figure 5g. The laser cut mold was made of a 3 mm thick transparent material. After all the sensors had cured, each side of the mold was separated for a convenient operation when removing it from the mold. As shown in Figure 5g, each side was assembled, and the mold was fixed with Kapton tape. Kapton tape was attached, without leaving air bubbles, to the mold using a stainless-steel ruler.

Figure 5h shows the acoustic window cured inside the mold. At this stage, because the receiving and transmitter do not exist inside the acoustic window, the temperature was set to 30 °C on the hot plate and cured for 24 h. As shown in Figure 5i, we created seven Rho-C pillars in the same manner. The completed Rho-C pillars were attached to all parts, except the part where the wire of the sensor came out. 

**Figure 6:**
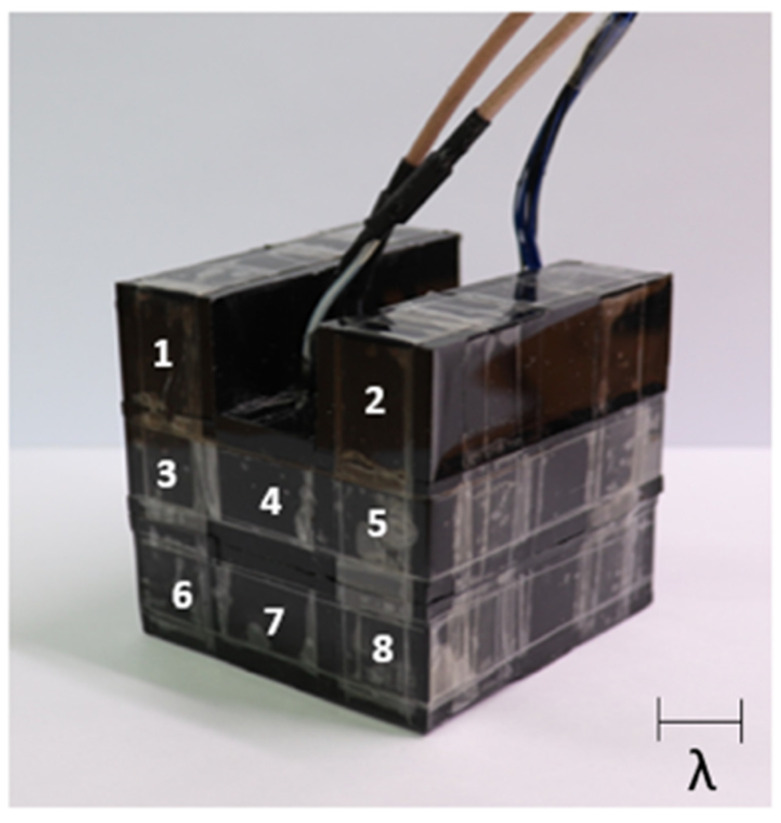
Fabricated transducer.

The appearance of the device that we finally completed is shown in Figure 6. As shown in the figure, the main device is located at the center (No. 4), and the remaining are made of Rho-C pillars. The purpose of the Rho-C pillars is to simulate the boundary condition of the main device when the device is operating in an array form.

## 5. Experimental Setup

**Figure 7:**
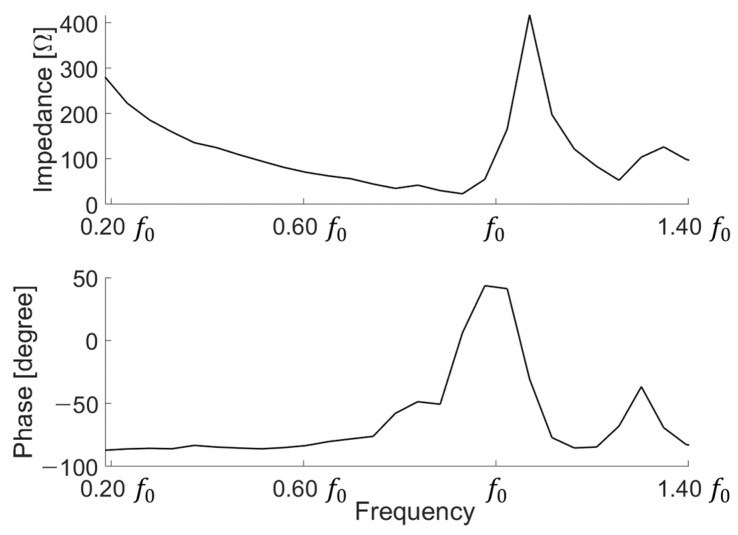
Measurement of electrical impedance.

To understand the characteristics of the fabricated device, we used an impedance analyzer (4194A impedance/gain-phase analyzer, Keysight, Santa Rosa, CA, USA) to determine the electrical characteristics. When using an impedance analyzer, the device was measured by floating the device from the floor to measure in a state where the device was not affected and without any effect. In Figure 7, the $f_{0}$ value of the fabricated device does not completely match the results of the simulation. It can be predicted that when we stack the devices to implement a low frequency, the alignment will not be perfectly matched, resulting in other modes of vibration that are different from the expected frequency range. Moreover, the fabricated device was higher than the magnitude of the simulation. In the case of a fabricated transducer, all electrodes are connected to each layer.

**Figure 8:**
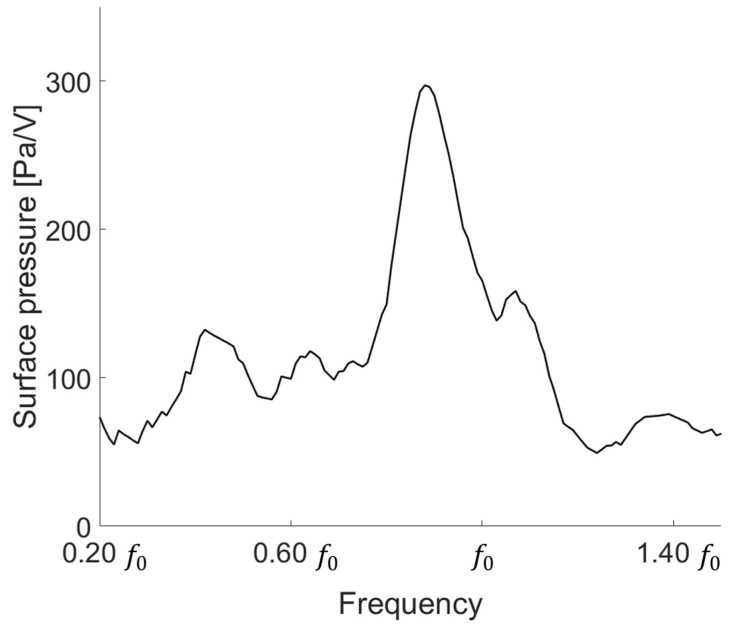
Surface pressure of the low-frequency transducer.

After measuring the electrical characteristics of the device, we conducted an experiment to determine the transmission characteristics. After applying 10 cycles of 300 mV to the device with a function generator (33500B Series, Keysight, Santa Rosa, CA, USA), a 40 dB amplifier (ultrasonic preamplifier, Olympus, Tokyo, Japan) was connected and measured with an oscilloscope (DSO1014A, Keysight, Santa Rosa, CA, USA). To determine the surface pressure of the low-frequency transducer, we measured from 0.20 $f_{0}$ to 1.40 $f_{0}$. The transducer showed the highest value at 0.81 $f_{0}$ at 297 Pa/V. As shown in Figure 8, the surface pressure was derived by applying the device area, loss, gain amplifier, and sensitivity of the low-frequency hydrophone. The actual measured output characteristic is less than the simulation result because our device has an acoustic window in front. Thus, the output is lower owing to the loss of the acoustic window. 

To investigate the techniques to reduce the reflected acoustic signal, we conducted an experiment in a water tank of 71.9 × 50.4 × 50.4 λ. All distances are indicated based on the wavelength of $f_{0}$. A mini water tank was used to conduct the experiment inside the lab; further, the low frequency was measured, and the wavelength was close to several centimeters. To reduce reflections as much as possible and make precise measurements, we attached a sound-absorbing material inside the water tank. The sound-absorbing material is 23 × 3.6 × 23 λ in size and has a wedge-shape structure. Double-sided tape was used to attach the sound-absorbing material to the inside of the water tank. A sound-absorbing material was also required at the bottom of the tank. The sound-absorbing material was relatively light, so it tended to rise above the water surface. Therefore, after adding 5 kg of dumbbells to each sound-absorbing material, we wrapped it with a transparent wrap. After measuring the electrical characteristics of the device, we conducted an experiment to determine the transmission characteristics. After applying 10 cycles of 300 mVpp to the device with function generator (33500B Series, Keysight, Santa Rosa, CA, USA), a 40 dB amplifier (Ultrasonic preamp, Olympus, Tokyo, Japan) was connected and measured with an oscilloscope (DSO1014A, Keysight, Santa Rosa, CA, USA). Measured after minimizing the reflected signal from the outside.

**Figure 9:**
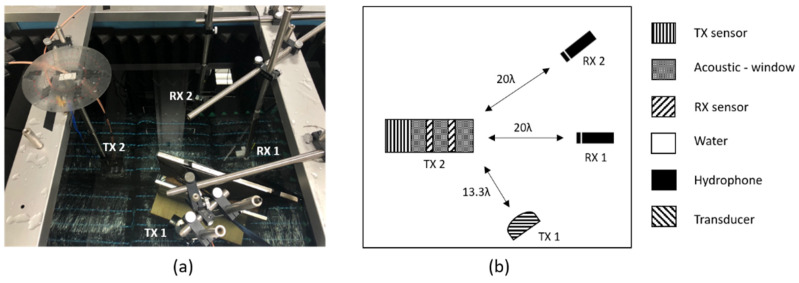
Experimental setup: (**a**) actual setup; (**b**) schematic of the experimental setup.

In Figure 9, we show how to measure and cancel the reflectance, which refers to the angle of incidence. Inside the water tank with the sound-absorbing material, we placed the proposed device on the left side and the low-frequency hydrophone (receiver, RX1 and RX2) on the right. TX1 is a commercial transducer used at low frequencies. Commercial transducers at low frequencies are not intended for immersion. The commercial transducer (TX1) was first connected to the Bayonet Neill-Concelman (BNC) cable, and then all the grooves of the BNC connection were filled with low-viscosity epoxy. After filling with epoxy, we proceeded for shielding as follows: A hole was made with a knife at the bottom of the disposable beaker to penetrate the cable through a disposable beaker. Subsequently, a commercial transducer to which a cable was connected was placed and degassed PDMS was poured. After curing at room temperature for one day and after validating the proper operation of the transducer, the experiment was conducted. 

When 0.33 $f_{0}$ 300 mV_pp_ was applied from the commercial transducer TX1, the signal could be measured by a hydrophone (RX1 and RX2). After finding the signal from the hydrophone and PVDF receiving sensor, we applied 0.33 $f_{0}$ 2.232 V_pp_ with the device TX2. At this time, the two transducers have different transmission sensitivities. Therefore, when transmitting, the amplitude must be different. TX1 and RX2 were located at the same angle. The phase was fixed at 180°. The experiment was conducted by changing the angle of the transducer. 

## 6. Results

**Figure 10:**
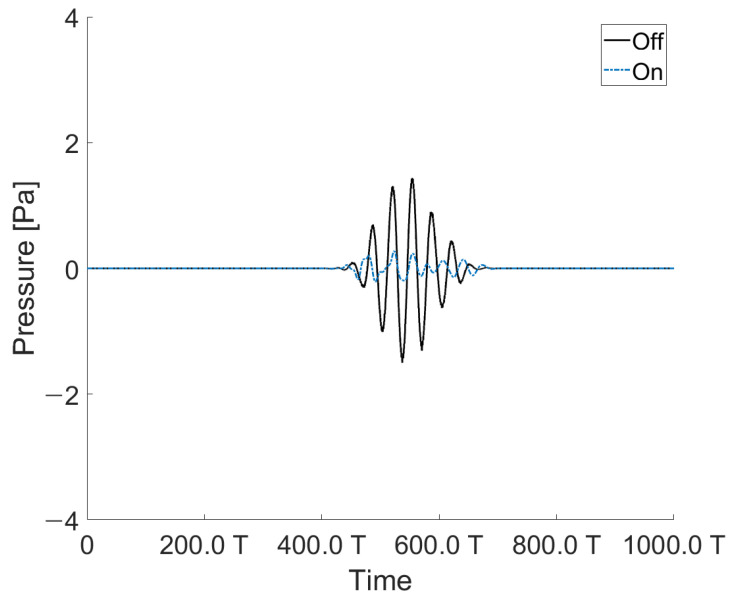
Acoustic signal reduction measured by a hydrophone located at the same angle as the angle of incidence (at 10°).

We conducted experiments and simulations to reduce the reflected sound of an incoming acoustic signal with a particular angle. It was arranged as follows to reduce the acoustic signal from a specific angle: a transducer capable of excitation with an acoustic signal at a specific angle (TX 1), a hydrophone (RX 2) positioned opposite to the same angle as TX1, and a transducer (TX 2) to reduce the reflected signal measured at RX2. A hydrophone (RX1) measures the acoustic signal going out perpendicularly from TX2. When the active control (TX 2) was activated and not driven, it was expressed as on and off, and the result is shown in Figure 10. In addition, signals other than the target reflections were detected because of reflections inside the water tank and water surface. The signals reflected from different surfaces overlapped with one another and merged with the signals of various frequency bands. To obtain sophisticated results, the signal was processed using a bandpass filter, and other signals were removed through a window, except for the target reflected signal.

The experiments were conducted to reduce the acoustic signal of the angle of incidence. The angle of incidence was 10–40° at intervals of 10°. Figure 10 shows that the reflected signal was reduced by −23 dB. Figure 10 shows the reflected signal when the angle of incidence was 10°.

**Figure 11:**
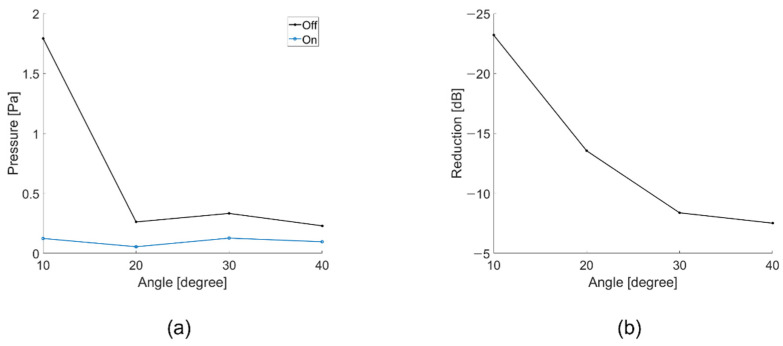
(**a**) Pressure depending on whether the controller is operating or not (at 10° to 40°); (**b**) Reduction ratio depending on whether the controller is operating or not (at 10° to 40°).

The acoustic signal was canceled at intervals of 10° from 10° to 40°. As shown in Figure 11b, it is expressed as the ratio between when the acoustic signal is controlled by our transducer and when the acoustic signal is applied with the angle of incidence from the outside. Accordingly, it shows the greatest effect at −23 dB at 10°. 

**Figure 12:**
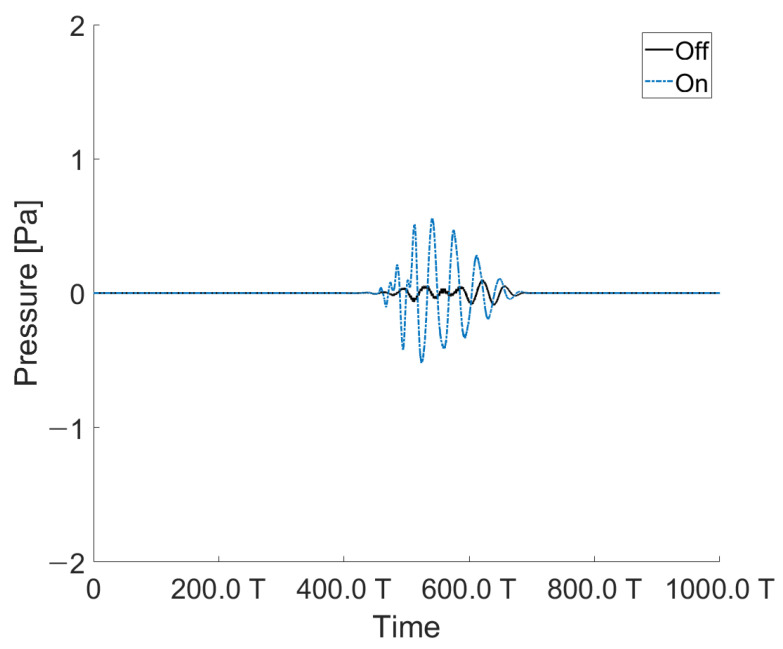
Acoustic signal measured by the hydrophone located in the perpendicular direction (RX 1) of the controller (at 10°).

**Figure 13:**
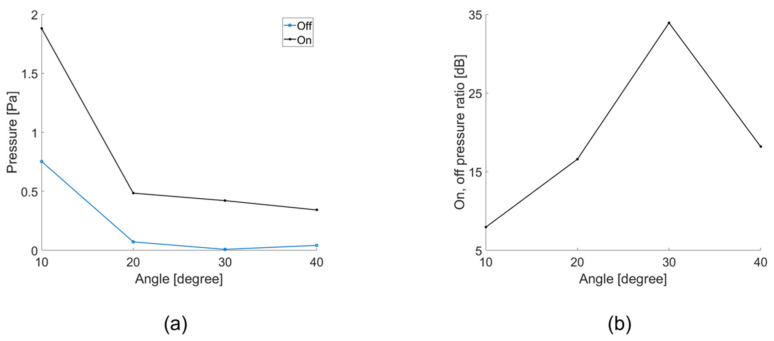
(**a**) Pressure measurements depending on whether the controller is operating or not (at 10° to 40°); (**b**) Reduction ratio depending on whether the controller is operating (hydrophone (RX 1) is located in the perpendicular direction of the transducer).

The angles of incidence and reflection were fixed at the same position at a certain angle. A transducer was placed on the angle of incidence, and a hydrophone was set on the reflection part. The controller was positioned between the angle of incidence and reflection. The signal detected by the hydrophone in the reflection part was controlled by the controller. Because the controller was oriented in the perpendicular direction, when the controller was operated to reduce the acoustic reflection signal located at the reflection angle, the signal in the perpendicular direction inevitably increased. The undesired acoustic signal was measured using an RX 1. An acoustic signal in the perpendicular direction is shown in Figure 12. When the controller was off, a small amount of acoustic signal was detected owing to the omnidirectional scattering from the structure. It worsened when the controller was on, as the controller was designed to compensate the reflecting signal at a specific angle. Figure 13 shows the perpendicular sound signal depending on whether the controller was driven.

If a single controller is used, the signal in the perpendicular direction will increase. However, the controllers were connected in parallel according to the pitch to form an array, and the signal going out in the perpendicular direction could be reduced. Signals coming in at the angle of incidence to the controllers arranged in parallel had a time delay based on the controller’s pitch. When all signals with a time delay were added together, the received signal was reduced. As a result, a parallel-connected controller should be implemented as a technique to reduce the reflected and perpendicular signals according to the angle of incidence. Hence, based on the experimental data, we simulated whether the pressure decreased in the perpendicular direction. The perpendicular pressures from 1 to 100 transducers were calculated. 

**Figure 14:**
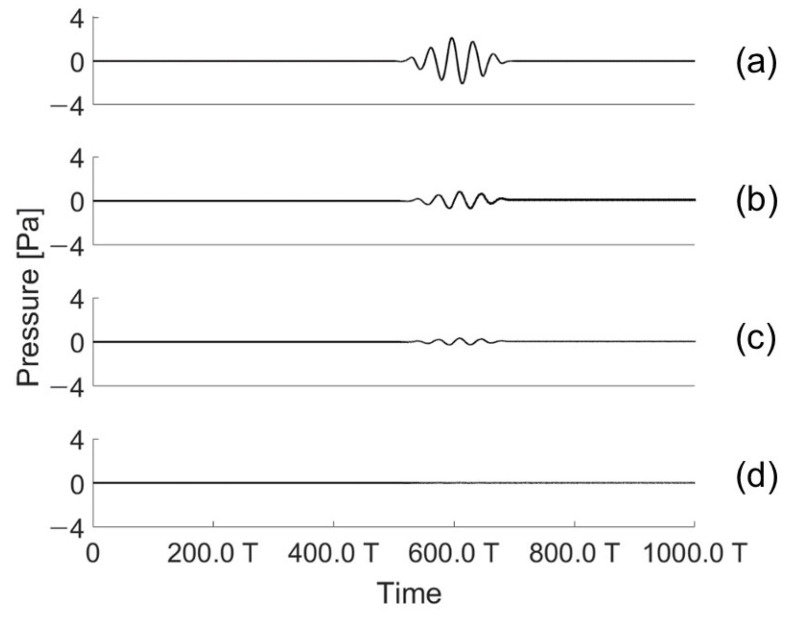
Acoustic signals emitted in the perpendicular direction when the array transducer is activated: (**a**) one; (**b**) two; (**c**) five; and (**d**) hundreds of transducers (at 30°).

After adding all the signals arriving at each device, the maximum value of the added value was extracted and calculated. As shown in Figure 14, as the number of transducers increased, the pressure in the perpendicular signal decreased. When there were more than two transducers, the signal going perpendicularly decreased significantly. Therefore, it is expected to be effective starting from three when implemented as an array.

**Figure 15:**
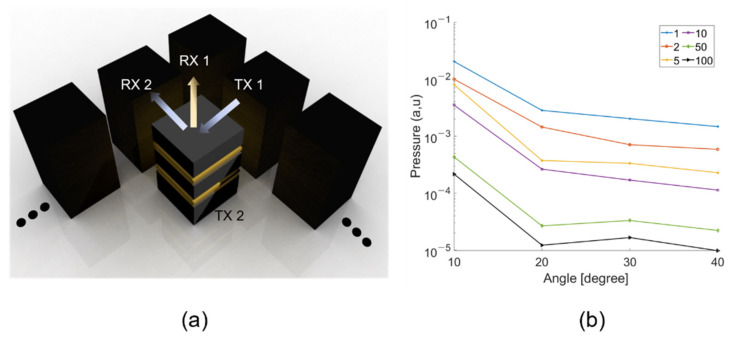
Perpendicular pressure referring to the number of transducers in an array. (**a**) array schematic (**b**) perpendicular pressure.

In Figure 14, the acoustic signals for 1, 2, 5, and 100 transducers when the angle of incidence is 30° are shown. All acoustic signals were calculated based on the data measured when driving a fabricated transducer. Delays were given considering the angle of incidence and the pitch between devices. This is the result of summing all the delayed signals to derive the maximum value. Therefore, in Figure 15, as the number of transducers increases, the signal emitted in the perpendicular direction clearly decreases. In Figure 15a, the amount emitted in the RX1 direction can be seen in Figure 15b. Thus, our proposed controller can control the reflection by referring to the angle of incidence, but when only one controller is driven, a perpendicular signal is emitted. Consequently, to effectively control the reflection according to the angle of incidence, the controller must be operated with an array. 

## 7. Discussion

We designed a transducer that can control reverberation based on the angle of incidence. The center frequency of the device, $f_{0}$ is three times of the operating frequency, 0.33 $f_{0}.$ If the device is designed to have 0.33 $f_{0}$ as the resonance frequency, the thickness of the piezoelectric material will become considerably thicker. To design the piezoelectric transducer for an ideal vibration mode, the width to thickness ratio should be more than 10:1. Therefore, the device is designed to be operated at a non-resonance region. In addition, the operation at the non-resonance mode will provide less ringing down effect, compared to the device operates at a resonance region. Referring to our previous study [42] comparing the resonance and non-resonance models of the device, the output performance of the resonance and non-resonance models is comparable.

An error exists between the characteristic simulation results and the actual measured value of the transducer. This is because the transducer was fabricated by stacking piezoelectric materials according to the target frequency. The alignment of the stacked piezoelectric materials was slightly mismatched; thus, the characteristics of the transducer were slightly different. The metal and epoxy intermediate layers for stacking would provide additional acoustic interference. 

We expressed the results by normalizing the time axis of the graph. In the case of acoustic research, several research papers presented normalized time and dimensions to generalize their works. It would give more general insight into the proposed methods. In our work, the dimensions were normalized to the wavelength which would provide better understanding of the device geometry. 

## 8. Conclusions

Research on sound reduction techniques in both air and water has been going on for a long time. Of them, we studied underwater acoustic reduction techniques. Although anechoic coatings and meta-structures are used for underwater acoustic reduction techniques, these methods are not effective for low-frequency applications. We chose to use a controller rather than using a meta-structure to reduce the reflected sound. Among the existing studies, the control of reflections for normal incidence has been studied, but it is difficult to find control of reflections with angles of incidence underwater, so this study was conducted. Experiments and simulations were conducted based on a transducer that can control the reflections and refer to the angle of incidence. If the designed transducer controls the reflected sound according to the angle of incidence, then the reflected sound that exists at the same position as the angle of incidence can be controlled. However, at this time, the sound wave is also spread omnidirectionally. The omnidirectional sound issue can be addressed using an array structure. By arranging the designed transducers in a row at an interval of half wavelength, the signal spreading in the perpendicular direction can be reduced. The proposed method can be applied to concealment techniques for moving objects in water, which are exposed to acoustic signals from various angles of incidence.

## Data Availability

Not applicable.
